# Avoiding Full Lymphadenectomies in Intermediate- and High-Risk Endometrial Cancer by Sentinel Lymph Node Biopsy Implementation

**DOI:** 10.3389/fonc.2021.654285

**Published:** 2021-04-15

**Authors:** Virginia García Pineda, Ignacio Zapardiel, Myriam Gracia, Jaime Siegrist, María Dolores Diestro, María Alonso, Alicia Hernández

**Affiliations:** Gynecological Oncology Unit, La Paz University Hospital, Madrid, Spain

**Keywords:** high-risk endometrial cancer, intermediate-risk endometrial cancer, early stage endometrial cancer, sentinel lymph node biopsy, systematic lymphadenectomy, isolated metastatic aortic lymph nodes

## Abstract

**Objective:**

To evaluate the role of sentinel lymph node biopsy (SLNB) to avoid staging lymphadenectomies by detecting nodal metastasis in intermediate- and high-risk endometrial cancer (EC).

**Methods:**

A single institutional retrospective study was performed including all patients with intermediate- and high-risk EC who underwent surgical nodal staging between January 2012 and December 2019. Patients with disseminated disease detected on imaging techniques or at the time of surgery were excluded. Patients were evaluable if they underwent nodal staging with SLNB and pelvic (PLD) and paraaortic (PALD) lymph node dissection. We analyzed the accuracy of the sentinel lymph node technique. Only patients with at least one sentinel lymph node (SLN) detected were included in the sensitivity and negative predictive value (NPV) analyses. The tracers used were technetium 99m, blue dye, and indocyanine green.

**Results:**

Eighty-eight patients presented intermediate- and high-risk EC (51 patients and 37 patients respectively) and underwent SLNB with consecutive PLD and PALD. The median (range) number of sentinel nodes retrieved was 2.9 (0–11). The global detection rate of SLN was 96.6% with a bilateral detection of 80.7% when considering all tracers used. However, when combination of indocyanine green and technetium was used the bilateral detection rate was 90.3%. Nodal metastases were detected in 17 (19.3%) cases, 8 (47%) of them corresponded to low volume metastasis (LVM), 7 (87.5%) of them diagnosed at ultrastaging pathologic exam. Finally, we obtained a sensitivity of 90%, a NPV of 97.5%, and a false negative rate (FNR) of 10% in the intermediate-risk EC compared to sensitivity of 85.7%, NPV of 96.6%, and FNR of 14.3% in the high-risk EC group. The only patient with isolated paraaortic nodal metastasis was found at the high-risk group, 1.1%.

**Conclusions:**

According to our results, full lymphadenectomy could be avoided by performing SLNB in patients with intermediate-risk EC because the only false negative case detected was at the beginning of ICG learning curve. For high-risk EC patients we did not find enough evidence to support the systematic avoidance of staging full lymph node dissection. Nevertheless, SLNB should be performed in all cases of EC as it improves LVM diagnosis substantially.

## Introduction

Endometrial cancer (EC) is the most common gynecological malignancy in developed countries, with an estimated incidence of 65,620 new cases in 2020, causing 12,590 deaths annually in the USA. Globally, 382,069 new cases of EC were diagnosed in 2018, with 89,909 deaths worldwide (1, 2).

Classic surgical staging of early-stage EC included pelvic and para-aortic lymphadenectomy in order to collect prognostic information and to guide the adjuvant treatment. However, the inclusion of systematic lymphadenectomy in the surgical treatment of EC has not showed any additional improvement in overall survival and disease-free survival of the patients while it increases perioperative morbidity (3, 4). Currently, complete lymphadenectomy is a standard surgical procedure in high-risk EC patients since 19% of these patients could present lymph node metastases (14% endometrioid histology and 32% non-endometrioid histology) (5).

Over the last decade, several clinical trials as SENTI-ENDO or FIRES (6, 7), showed that SLN biopsy seems to be as accurate as systematic lymphadenectomy to evaluate the nodal status of early-stage EC reporting a high sensitivity and negative predictive value to detect nodal involvement (84 and 97% *vs* 97.2 and 99.6%, respectively (6–8). These studies included mostly no high-risk disease for lymph node involvement which could influence the false negative rate.

Some studies reported a false negative rate in SLNB in EC ranging from 5 to 20% among high-risk patients (9, 10). The main drawback of SLNB technique in high-risk tumors is the lack of para-aortic assessment so isolated lymph node metastases would not be detected. In addition, the cervical injection of the tracer (the most extended method) would prevent from para-aortic drainage through the infundibulopelvic ligament.

Soliman et al. evaluated the accuracy of SLNB in high-risk EC reporting a sensitivity of 95% and a FNR of 4.3%, which developed an update in several clinical guidelines including the SLNB in the standard management of endometrial cancer (10).

The last National Comprehensive Cancer Network (NCCN) guideline and the recent update of the European guideline of Gynecological Oncology include the use of SLNB in high-risk EC as a reasonable option (11, 12).

The aim of our study was to evaluate the role of sentinel lymph node biopsy (SLNB) to avoid staging lymphadenectomies by detecting nodal metastasis in intermediate- and high-risk endometrial cancer.

## Material and Methods

We carried out a retrospective single-institutional study that included all consecutive patients initially diagnosed of intermediate- and high-risk EC and treated at our institution between January 2012 and December 2019. Data were collected from the medical records after Institutional Review Board approval (#PI-3846). All women with presumed intermediate- or high-risk EC by European risk classification (12) and Federation of Obstetrics and Gynecology (FIGO) stage I-II were assessed for eligibility. Intermediate-risk cases were defined as those endometrioid histotypes presenting ≥50% myometrial invasion and histological grade 1–2; or <50% of myometrial invasion and histological grade 3. High-risk cases were defined as those endometrioid histotypes presenting cervical stromal involvement; or ≥50% of myometrial invasion and histological grade 3; or high-risk histology including serous, clear cell, and carcinosarcoma tumors based on preoperative biopsy.

All patients underwent a preoperative imaging work-up with vaginal ultrasound or MRI to evaluate myometrial and cervical invasion. CT-scan or PET/CT was performed in high-risk cases in order to exclude nodal involvement or metastatic disease. Patients with suspected disseminated or locally advanced disease were excluded.

All patients included underwent total hysterectomy, bilateral salpingo-oophorectomy, SLNB, PLD, and PALD up to the left renal vein level in most of cases. Patients where SLN mapping or complete PLD and PALD were not performed were excluded from the study. In addition, patients with peritoneal disease or nodal macroscopic involvement identified intraoperatively were also excluded from the study.

Our SLNB protocol included the next steps: Firstly, the day before surgery two cervical injections at 3 and 9 o’clock (5 mm superficial and 15 mm deep) of 2 ml of technetium sulfur colloid (Tc99) were administered with a 25-gauge spinal needle (13). Lymphoscintigraphy images were obtained 2 h after the injections with the integration of single-photon emission computed tomography (SPECT/CT); Second, intraoperatively, 4 ml of methylene blue or indocyanine green (ICG) dilution 2.5 mg/ml were injected in the same location that technetium (2 ml per side, 5 mm superficial and 15 mm deep); Third, during surgery all the pelvic areas were carefully inspected for lymph ducts, following the main lymphatic drainage pathways. Lymph nodes marked by technetium (hot lymph nodes) and/or those marked by ICG/blue were identified and removed.

The tracers used during the study period were: from January 2012 until October 2014 Tc99 + methylene blue; from October 2014 until December 2018 Tc99 + ICG; and finally, from 2019 ICG alone has been used as single tracer.

All sentinel lymph nodes were ultrastaged postoperatively by multiple sectioning at 200 μm intervals. Each section was also divided at 50-μm intervals and stained with hematoxylin and eosin. An additional slide of each interval was used for an immunohistochemistry exam (IHC) with an anticytokeratin antibody dilution (cytokeratins AE1–AE3). Non-sentinel lymph nodes were evaluated by routine sectioning and H&E staining. Lymph node status was defined using the criteria of American Joint Committee on Cancer for breast cancer (2002): Isolated tumor cells (ITCs) were defined as a focus of metastatic disease measuring ≤0.2 mm; micrometastasis (MIC) was defined as a focus of metastatic disease between >0.2 and 2 mm; and macrometastasis (MAC) was defined as a focus of metastatic disease >2 mm (14). Those lymph nodes without tumor present on pathologic evaluation were reported as negative and lymph nodes with MAC, MIC, or ITCs were considered to be positive on final pathology. LVM was defined as ITCs and MIC together.

An analysis of diagnostic test was performed including the sensitivity, false negative rate (FNR), and negative predictive value (NPV) of SLNB, considering the gold standard the complete lymphadenectomy. Only patients with at least one sentinel lymph node detected were included in the sensitivity and negative predictive value analyses comparing to final pathology. We also estimated overall and bilateral detection rates among intermediate- and high-risk patients, considering the bilateral detection rate according to the tracer used. The sensitivity of SLNB was described as the proportion of patients with node-positive disease with successful SLN mapping who had metastatic disease correctly identified in the sentinel lymph node.

The overall detection rate was defined as the proportion of patients in which at least one SLN was identified. False negative rate was defined as the proportion of cases with negative bilateral SLNB and positive non-SLN at final pathology. Qualitative variables were reported with absolute numbers and percentages. Quantitative variables were reported as median and range. Categorical variables were compared using the chi-square test for univariate analysis. All statistical analysis were performed using the software SPSS Statistics v.24.0 (IBM Corp., Armonk, NY, USA).

## Results

**Figure 1:**
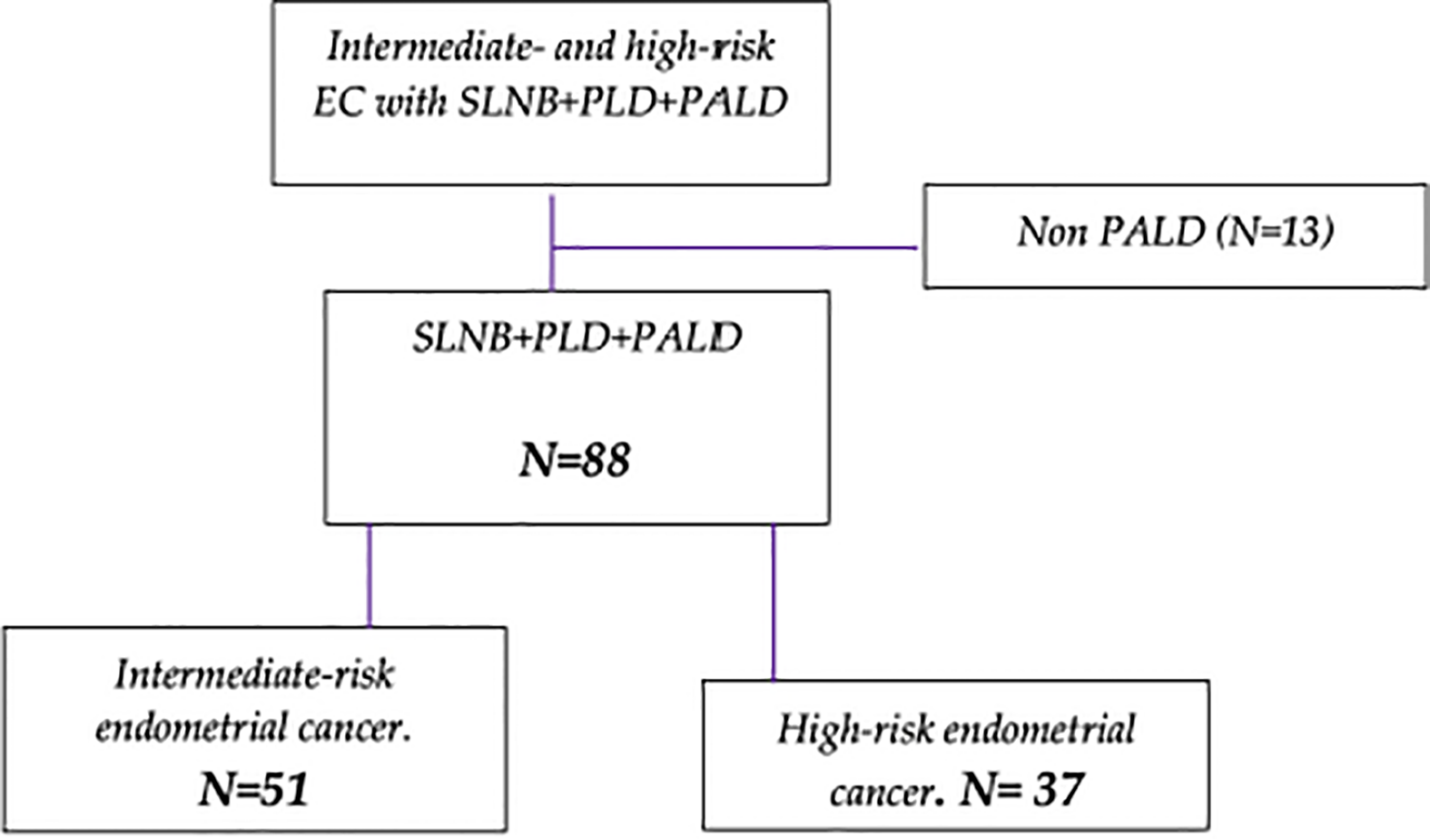
Enrollment of patient diagram. (SLNB = Sentinel lymph node biopsy. PLD = Pelvic lymph node dissection. PALD = Paraaortic lymph node dissection).

Flow diagram of patient inclusion is showed in **Figure 1**. A total of 101 cases of intermediate- and high-risk were enrolled, among them, 88 cases of endometrial cancer were evaluable and included in the study, 51 cases of intermediate-risk and 37 cases of high-risk endometrial cancer.

Demographic and final clinicopathological features are summarized in **Table 1**. The majority of patients, 86 cases (97.7%), were operated by laparoscopic approach and extraperitoneal paraaortic approach was performed in 65 cases (73.9%). The upper border of PALD was the left renal vein in 81 patients (92%), in the remaining 7 cases (8%) the dissection was up to inferior mesenteric artery (IMA) due to intraoperative complications. On final pathology 69 patients (78.4%%) presented early stage and advanced stage was presented in 19 patients (21.6%%) being the most frequent histology endometrioid, 61 patients (69.3%%), and serous, 15 cases (17%), adenocarcinoma (**Table 1**).

**Table 1:** Demographic characteristics of the study population.

Variables	N
**Age (years)** Median (range)	66 (45–85)
**BMI** Median (range)	28 (17–40)
**Histology**	
Endometrioid	61 (69.3%)
Serous	15 (17%)
Clear cell	6 (7%)
Carcinosarcoma	6 (7%)
**Grade**	
G1	24 (27.3%)
G2	28 (31.8%)
G3	36 (40.9%)
**Surgical approach**	
Laparoscopy	86 (97.7%)
Laparotomy	2 (2.3%)
Extraperitoneal PALD	23 (26.1%)
Transperitoneal PALD	65 (73.9%)
**Upper border of PALD**	
Left renal vein	81 (92%)
Inferior mesenteric artery	7 (8%)
**SLN mapping**	88(100%)
**FIGO stage**	
IA	35 (39.7%)
IB	31 (35.2%)
II	3 (3.4%)
IIIA	2 (2.3%)
IIIC1	11 (12.5%)
IIIC2	6 (6.8%)

BMI, body mass index; PLD, pelvic lymph node dissection; PALD, paraaortic lymph node dissection.

At least one SLN was retrieved in 85 cases being overall SLN detection rate 96.6%. Bilateral pelvic detection rate was achieved in 71 cases (83.5%). When we analyzed the data in each risk group, the bilateral detection rate in the intermediate-risk group was 81.6% and in the high-risk group was 86.1%. Regarding the use of different tracers, combination of Tc99 with blue dye has been used in 40 patients (47.1%), combination of Tc99 and ICG in 39 cases (45.9%), and ICG in 6 patients (7.1%) Bilateral detection rates based on tracer used are shown in **Table 2**.

**Table 2:** Sentinel lymph node unilateral and bilateral detection rates with different tracers.

Tracer used	Unilateral detection rate N (%)		Bilateral detection rate N (%)	N (%)	
ICG+Tc99	5 (12.8)	34 (87.2)		39 (45.9)	
Tc99+Blue dye	9 (22.5)	31 (77.5)		40 (47.1)	P = 0.27
ICG	0 (0)	6 (100)		6 (7.1)	
Overall detection	14(16.5)	71 (83.5)		85(100)	

**Figure 2:**
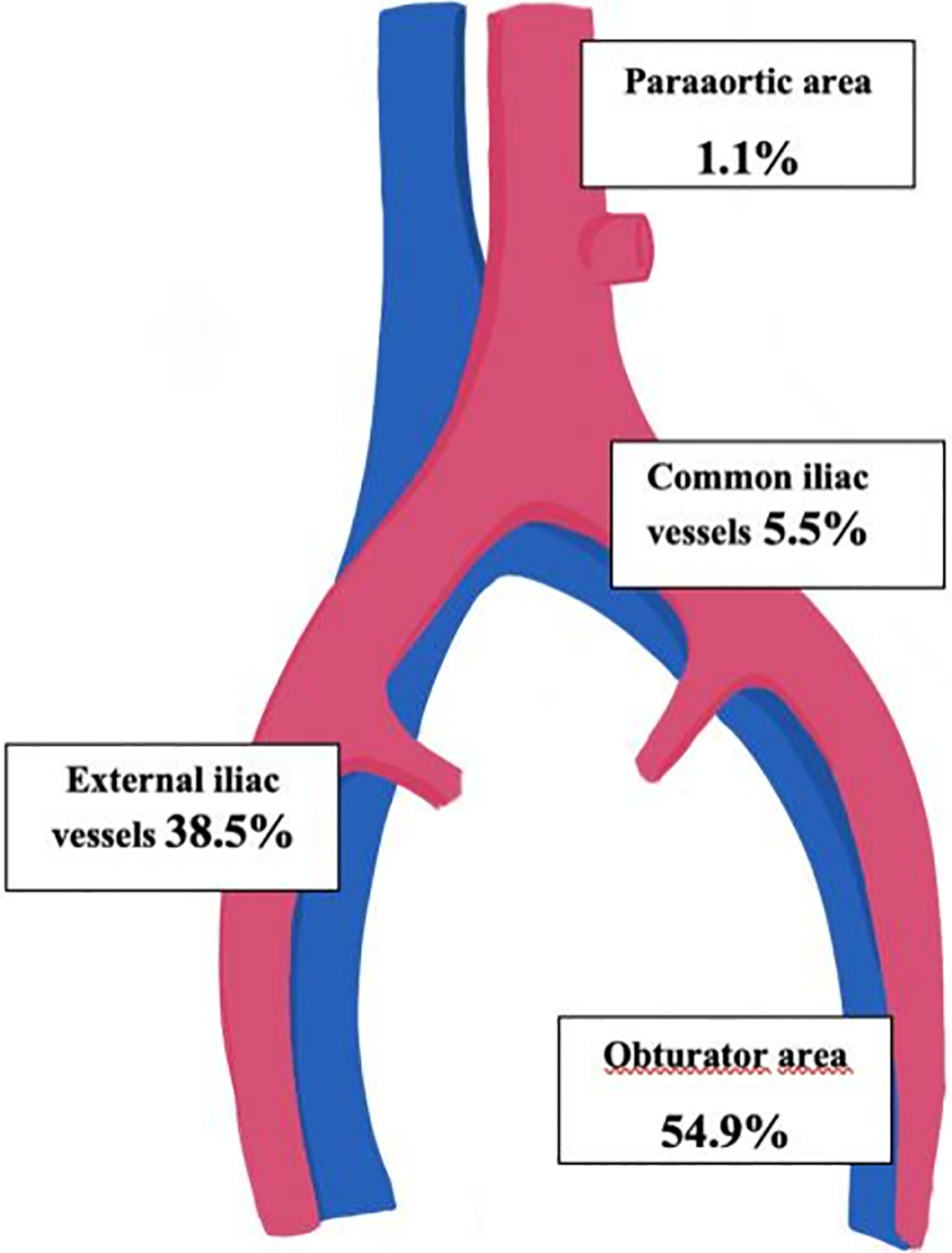
Anatomic distribution of sentinel lymph nodes.

Among 85 cases in which SLN was detected, a total of 251 SLN were removed. The median number of SLN retrieved was 2.9 (range 0–11) per patient and the median numbers of pelvic and paraaortic nodes were 14.7 (range 4–36) and 16.7 (range 2–39) respectively. The anatomical distribution of SLN was as follows: 54.9% in obturator area, 38.5% in external iliac vessels area, 5.5% in iliac common vessels area, and finally 1.1% in paraaortic area (**Figure 2**). There were 15 (17.6%) cases with positive SLN with the following metastasis distribution: 5 (37.5%) corresponded to isolated tumor cells, 3 (12.5%) to micrometastasis, and 7 (50%) to macrometastasis.

On final pathology there were 17 patients (19.3%) with nodal metastatic disease, among them, the only positive node was the SLN in 10 patients (58.8%). Concerning LVM disease, 4 (23.5%) patients presented MIC and 4 (23.5%) patients presented ITCs, 7 (87.5%) of them diagnosed at ultrastaging pathologic exam. Finally, 2 patients (11.8%) presented nodal disease in non-SLN with negative SLN at final pathology, therefore, considered as two false negative cases. Details of all nodal involved cases are included in **Table 3**.

**Table 3:** Nodal metastatic disease distribution.

Presurgical FIGO Stage	Grade	Histology	Status SLN	Status pelvic nodes	Status paraaortic nodes
II	1	Endometrioid	MAC	–	–
IB	2	Endometrioid	MIC	–	–
IB	3	Endometrioid	MIC	–	–
IB	2	Endometrioid	MIC	–	–
IB	1	Endometrioid	MAC	–	–
IB	1	Endometrioid	MIC	–	–
II	2	Endometrioid	ITCs	–	–
IB	3	Serous	MAC	MAC	MAC
IA	2	Endometrioid	–	MIC	–
IB	1	Endometrioid	MAC	–	MAC
II	3	Serous	MAC	MAC	MAC
IB	2	Endometrioid	MAC	–	–
IB	3	Serous	ITCs	–	MAC
IB	1	Endometrioid	MIC	–	–
IB	1	Endometrioid	ITCs	–	–
IB	2	Clear cells	MAC	MAC	MAC
IB	3	Clear cells	–	–	MAC

MAC, macrometastasis; MIC, micrometastasis; ITCs, isolated tumor cells; - = negative nodes.

When we analyzed the accuracy of SLNB by groups we obtained a sensitivity of 90%, NPV of 97.5%, and FNR of 10% in the intermediate-risk EC group. When we studied the accuracy of SLNB in this group in the last 4 years, the sensitivity and NPV increased up to 100% with 0% of false negative rate. Whereas in the high-risk EC group, we observed a sensitivity of 85.7%, NPV of 96.6%, and FNR of 14.3%. The only case of isolated para-aortic lymph node metastasis found was the unique false negative case in the high-risk group.

On the other hand, 71 patients (80.7%) presented negative nodes, corresponding to practically half of each group of patients. In the intermediate risk group, 41 (80.4%) patients presented negative nodes being also negative in 30 (81.1%) patients in the high-risk group.

The three patients (two cases of intermediate-risk and one of high-risk) for whom no SLNs were identified went on to full lymphadenectomy, none of the additional nodes evaluated were malignant.

## Discussion

The inclusion of SLNB would avoid the performance of complete lymphadenectomies which have not shown any impact on the survival of patients with early stage of EC according to two randomized clinical trials [ASTEC trial (2009), Benedetti et al. trial, (2008)] (15, 16).

In the last decade, SLN biopsy has obtained enough scientific evidence to relegate complete lymphadenectomy to the past, demonstrating the same oncological accuracy in nodal staging in early stages of endometrial cancer. The most important clinical trial to validate the accuracy of SLNB, FIRES trial, included only 28% of high-grade histology in the study population (6). The main criticism of these studies was the low percentage of high-grade histology included which has the highest risk of metastasis and isolated paraaortic disease. Therefore, despite recent SGO recommendations on the inclusion of SLN biopsy in early stages of EC (17), the role of the technique in high-risk disease remains controversial.

In this retrospective study of SLNB, we analyzed the accuracy of the technique including that cases in which at least one SLN was identified. The global detection rate of SLN was 96.6% with a bilateral detection rate of 83.5% in accordance with the literature (18). While our study was not able to demonstrate significant differences in detection rates by mapping technique, ICG is associated with higher detection rate and bilateral rate according with previous literature (8).

In our validation analyses of SLNB in patients with intermediate- and high-risk EC, this technique detected 90 and 85.7% of patients with positive nodes respectively. Our study demonstrated in the intermediate-risk group, high sensitivity and NPV with only one false negative case identified at pelvic level. This case occurred during the early learning curve of ICG mapping and probably it had an impact on our SLN mapping accuracy. On the other hand, no cases of isolated paraaortic metastasis were identified in this group. With the improvement of our learning curve in SLN mapping, no more false negative cases were recorded during the last 4 years. Therefore, the inclusion of SLN biopsy in this group could be considered following the NCCN surgical SLN algorithm as it has demonstrated good accuracy and a false negative rate <5% in the detection of nodal metastases in recent prospective studies (10, 19).

However, in the high-risk group the sensitivity and NPV dropped slightly to 85.7 and 96.6% respectively. Concerning false negative rate, it increases up to 14.3%, less compared to other retrospective series described in the literature with up to 20% false negative rate (9).

Nevertheless, more recent prospective and retrospective studies have demonstrated more promising results on this subject. The retrospective study by Touhami et al. (2017) described a sensitivity and NPV of 95.8 and 98.2% respectively (19) and Holloway et al. (2017) reported on a prospective study a sensitivity of 97.5%, a NPV of 99.3%, and a FNR of 2.5% applying SLN mapping with different tracers in intermediate- and high-risk EC (18).

The retrospective study of Papadia et al. (20) aimed to validate the laparoscopic ICG SLN mapping in patients with grade 3 or high-risk histology. This group reported 23.8% of Lymph node metastasis with only one false negative case which corresponded to a metastatic non-SLN isolated para-aortic metastasis, according with our results in high-risk group. This study showed a sensitivity, FNR, and NPV of 90, 10, and 97.1% respectively which is consistent with our results (20).

In the last year, two large prospective studies on this topic were published. SHREC trial by Persson et al. (21) included 275 patients with intermediate- and high-risk EC who underwent SLN biopsy followed by robot-assisted pelvic and para-aortic lymphadenectomy (in 81% of cases). The analyses reported a sensitivity of 98% and a NPV of 99.5% applying surgical SLN algorithm with ICG. Two cases of false negative SLN were detected in the analyses. The authors concluded that when SLN algorithm was performed by experienced surgeons, it has the potential to safely replace lymphadenectomy in these cases of EC without the need for para-aortic LND (21).

Recent publication of SENTOR study by Cusimano et al. (22), described a SLNB sensitivity of 96% and a NPV of 99% with only one false negative case. Comparing this study with ours, we identified one more false negative case, one of them an isolated paraaortic metastasis. We should consider that 100% of our population underwent PALD while in SENTOR and in SHREC studies PALD was performed in 80 and 81% of their population respectively (22).

The main strength of our study is that all patients included presented a comprehensive surgical staging based on SLNB, PLD, and PALD, which allowed us to accurately define the sensitivity, NPV, and FNR of SLNB in intermediate- and high-risk EC.

Another important consideration of SLN biopsy in high-risk EC is the proportion of patients who present additional non-SLN metastasis in the presence of metastatic SLN because these patients could benefit from complete lymphadenectomy. The recent study of Taskin et al. (23) evaluated the feasibility of SLN mapping in uterine confined endometrial cancer, it reported 60% of patients with macrometastatic SLN who also presented non-SLN involvement. All of these patients received chemotherapy but there is not consistent evidence suggesting that leaving these nodes *in situ* has a detrimental effect on survival (23). Retrospective studies of Buda et al. (24) evaluated the impact of SLN mapping compared to SLN plus complete lymphadenectomy on the prognosis in patients with intermediate and high-risk EC and concluded that the 5-year recurrence free survival was similar in both groups (79.2 *vs* 81.6 respectively, p = 0.831) (24, 25). Therefore, the most important concept in high-risk EC is achieve an adequate nodal staging in order to target adjuvant therapy properly.

Moreover, high-risk EC has the highest risk for metastasis and isolated paraaortic metastasis, the only false negative case in our high-risk population was an isolated paraaortic metastasis. In order to improve this lack, the study of Ruíz et al. (26) included dual cervical and fundus injection of ICG in SLNB with aortic SLN detected in 59.5% of cases (26).

A further important concept of SLN biopsy is that ultrastaging of pelvic SLN nodes decreased the true prevalence of isolated paraaortic dissemination with LVM detection. The study of Multinu et al. (27) showed that ultrastaging of pelvic nodes reduced by 30% true isolated paraaortic metastasis prevalence identifying occult LVM (27). In our study, 47% of LNM corresponded to LVM, among them, 87.5% were only detected at SLN ultrastaging.

In conclusion, our study gives another argument for the inclusion of SLNB in surgical staging of intermediate- and high-risk endometrial cancer. SLN biopsy seems to be an accurate alternative to systematic lymphadenectomy in patients with intermediate-risk endometrial cancer after improvement of our learning curve with the new tracer. On the contrary, in high-risk endometrial cancer we would need to improve the false negative rate of the technique to be able to avoid systematic lymphadenectomy. A proper accuracy of SLNB was not achieved in this group, probably by the low number of cases included.

Nevertheless, SLN mapping should be included as part of nodal staging in both intermediate- and high-risk disease since it increases the overall detection of nodal metastasis when compared to routine systematic lymphadenectomy. Although SLNB ultrastaging has shown an increase in the low volume metastasis detection, its oncological impact remains controversial.

## Data Availability Statement

The data analyzed in this study is subject to the following licenses/restrictions: patient data are protected and cannot be disclosed. Requests to access these datasets should be directed to VP, virginiagarciapineda@gmail.com.

## Ethics Statement

The studies involving human participants were reviewed and approved by Institutional Review Board approval (#PI-3846). Written informed consent for participation was not required for this study in accordance with the national legislation and the institutional requirements.

## Author Contributions

VP contributed to designing the study, analyzing the data, and writing the manuscript. IZ and AH contributed to the writing of the manuscript. The rest of the authors contributed in data collection. All authors agree to be accountable for the content of the work. All authors contributed to the article and approved the submitted version.

## Funding

This study has been funded by Instituto de Salud Carlos III through the project "PI20/01368" (Co-funded by European Regional Development Fund) Project "PI20/01368", funded by Instituto de Salud Carlos III and co-funded by European Union (ERDF) Funding: ISCIII ("PI20/01368"), co-funded by ERDF, "A way to make Europe".

## Conflict of Interest

The authors declare that the research was conducted in the absence of any commercial or financial relationships that could be construed as a potential conflict of interest.
